# Case Report: Biallelic Variant in the tRNA Methyltransferase Domain of the AlkB Homolog 8 Causes Syndromic Intellectual Disability

**DOI:** 10.3389/fgene.2022.878274

**Published:** 2022-04-28

**Authors:** Ahmed Waqas, Anam Nayab, Shabnam Shaheen, Safdar Abbas, Muhammad Latif, Misbahuddin M. Rafeeq, Ibtesam S. Al-Dhuayan, Amany I. Alqosaibi, Mashael M. Alnamshan, Ziaullah M. Sain, Alaa Hamed Habib, Qamre Alam, Muhammad Umair, Muhammad Arif Nadeem Saqib

**Affiliations:** ^1^ Department of Zoology, Division of Science and Technology, University of Education, Lahore, Pakistan; ^2^ Microbiology and Biotechnology Research Lab, Department of Biotechnology, Fatima Jinnah Women University, The Mall, Rawalpindi, Pakistan; ^3^ Department of Higher Education, Government Girls Degree College Lakki Marwat, Lakki Marwat, Pakistan; ^4^ Department of Biological Science, Darmouth College, Hanover, NH, United States; ^5^ Department of Pharmacology, Faculty of Medicine, Rabigh, King Abduaziz University, Jeddah, Saudi Arabia; ^6^ Department of Biology, College of Science, Imam Abdulrahman bin Faisal University, Dammam, Saudi Arabia; ^7^ Department of Microbiology, Faculty of Medicine, Rabigh, King Abduaziz University, Jeddah, Saudi Arabia; ^8^ Department of Physiology, Faculty of Medicine, King Abduaziz University, Jeddah, Saudi Arabia; ^9^ Medical Genomics Research Department, King Abdullah International Medical Research Center (KAIMRC), King Saud Bin Abdulaziz University for Health Sciences, Ministry of National Guard Health Affairs (MNGH), Riyadh, Saudi Arabia; ^10^ Department of Life Sciences, School of Science, University of Management and Technology (UMT), Lahore, Pakistan; ^11^ Department of Medical Laboratory Technology, National Skills University Islamabad, Islamabad, Pakistan

**Keywords:** missense variant, biallelic variant, whole exome sequencing, tRNA methyl transferase, intellectual disability, posttranscriptional modification

## Abstract

Intellectual disability (ID) has become very common and is an extremely heterogeneous disorder, where the patients face many challenges with deficits in intellectual functioning and adaptive behaviors. A single affected family revealed severe disease phenotypes such as ID, developmental delay, dysmorphic facial features, postaxial polydactyly type B, and speech impairment. DNA of a single affected individual was directly subjected to whole exome sequencing (WES), followed by Sanger sequencing. Data analysis revealed a novel biallelic missense variant (c.1511G>C; p.(Trp504Ser)) in the *ALKBH8* gene, which plays a significant role in tRNA modifications. Our finding adds another variant to the growing list of ALKBH8-associated tRNA modifications causing ID and additional phenotypic manifestations. The present study depicts the key role of the genes associated with tRNA modifications, such as *ALKBH8*, in the development and pathophysiology of the human brain.

## Introduction

ALKBH8 (Alkylated DNA Repair Protein AlkB Homolog 8) is a nuclear and cytosolic protein essential for tRNA modifications, for normal survival after DNA damage, can inhibit apoptosis by helping boost cell survival and angiogenesis (Fu et al., 2010; Songe-Møller et al., 2010). There are eight different members of the AlkB family that have been identified in humans (ALKBH1–ALKBH8) and mice (Alkbh1–Alkbh8) (Aravind and Koonin, 2001; Kurowski et al., 2003; Gerken et al., 2007). Most ALKBH proteins have unknown functions, while ALKBH8 differs from the other versions by two annotated protein domains. The ALKBH8 protein contains three main domains, namely, an N-terminal RNA recognition motif (RRM), a C-terminal S-adenosyl-L-methionine (SAM)–dependent methyltransferase (MTase) motif, and a central conserved AlkB oxygenase domain. The MTase and AlkB-like domains have been reported to covalently catalyze hypermodifications of the wobble nucleotide base in specific tRNAs (Fu et al., 2010).

ALKBH8 performs the final methylation step during the formation of the wobble uridine modification mcm5U in mammals. The MTase domain catalyzes the methyl esterification of modified wobble uridine (U34) residues, resulting in the formation of mcm5U (5-methoxycarbonyl methyluridine) and mcm5s2U (5-methoxycarbonyl methyl-2-thiouridine) (Tsujikawa et al., 2007; van den Born et al., 2011). The mcm5U is a common modification intermediate for thiolation and ribose methylation (Kaneko et al., 2003).

Furthermore, ALKBH8 also interacts with TRM112 forming a heterodimeric complex, which aids in catalyzing the methyl esterification forming mcm5U and mcm5s2U. The mcm5Um is involved in the selenoprotein synthesis, and thus, a reduced level of Gpx1 (glutathione peroxidase 1) was observed in the *Alkbh8* ^−/−^ mice (Songe-Møller et al., 2010). Gpx1 is a selenium-containing enzyme that reduces hydrogen peroxide and alkyl hyperoxides, thus protecting against traumatic brain injury (TBI). Using transgenic knockout mice, overexpression of Gpx1 was observed in many organs, such as the brain. The Gpx1 deficient mice further revealed that this enzyme enhances neuroprotection in response to the oxidative challenge. The brains of *GPx1* ^*−/−*^ mice were reported to be more vulnerable to ischemia/reperfusion, cold-induced brain injury, and mitochondrial toxin treatment. The *GPx1* ^*−/−*^ mice also exhibited neurological deficits (Pitts et al., 2014).

This study illustrates that a novel homozygous missense variant in the *ALKBH8* results in severe syndromic ID. The present investigation expands the mutational spectrum of ALKBH8-associated ID.

## Materials and Methods

### Ethical Appraisal

**FIGURE 1:**
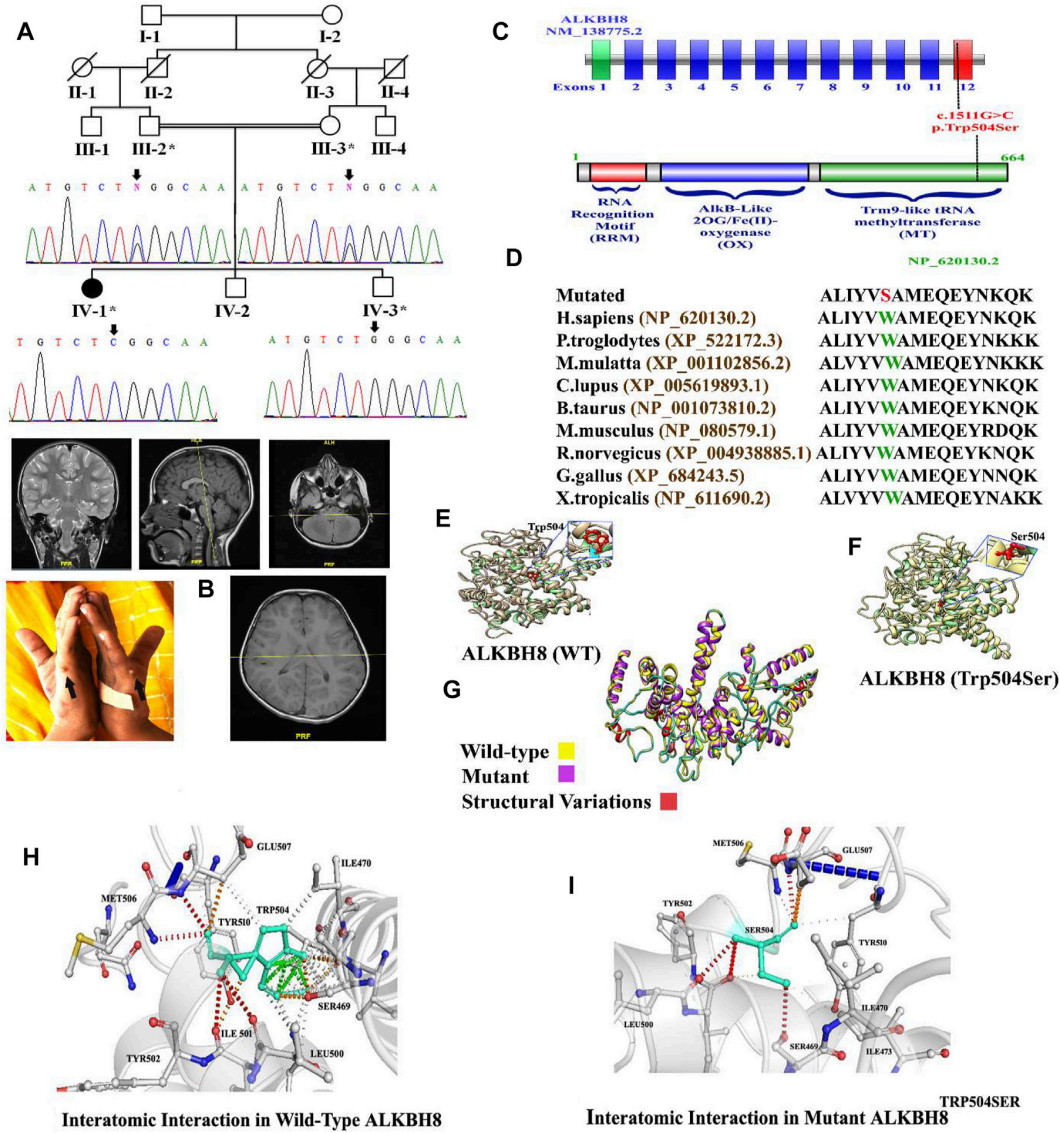
**(A)** Pedigree of the investigated family. Sanger sequencing electrograms shown below each member. **(B)** Brain MRI of the index (IV-1) and bilateral postaxial polydactyly type B. **(C)** Schematic representation of the *ALKBH8* exons and protein domain. Dotted line represents the variant identified in the present study and its location in the exon and domain. **(D)** Partial amino acid sequence of ALKBH8 protein showing conservation of Trp504 amino acid across different species. **(E–I)** 3D protein modeling comparison of the ALKBH8 wild-type and mutated proteins.

The present study was approved by the institutional review board of the University of Education (UOE), Lahore, Pakistan, and followed Helsinki protocols. Written informed consents for the publication of this case, photographs, and related data were obtained from the parents. In addition, a pedigree was constructed (Figure 1A), and the peripheral blood samples were obtained from all available normal and affected individuals of the family.

### Genomic DNA Extraction

The genomic DNA was extracted from the fresh blood of all the available members of the family using the QIAamp DNA Micro kit using standard procedures and was quantified using NanoDrop™ spectrophotometer using standard methods (Younus et al., 2019).

### Whole Exome Sequencing)

DNA of the single affected individual (IV-1) was subjected to WES using the Agilent SureSelect target enrichment workflow from the created DNA library. WES was performed using the Illumina work system with a minimum coverage of 20× of 98% of the total reads (Asiri et al., 2020; Umair et al., 2020; Umair et al.,2021). After sequencing, the sequence was aligned to the reference human genome build UCSC hg19 reference system [GRCh38/hg38]. An online Illumina data analysis tool (BaseSpace, Illumina; https://basespace.illumina.com) was used to analyze the patient VCF to identify the culprit gene/variant.

### Sanger Sequencing

The identified candidate variant was Sanger sequenced bidirectionally in all the available members of the family. Sanger sequencing was performed as described earlier (Umair et al., 2019). Primer sequences were designed using the Primer3 online software (http://frodo.wi.mit.edu/primer3/) and will be provided upon request.

### Pathogenicity Index

The pathogenicity variant was calculated via different online software: MutationTaster, VarSome, PolyPhen-2, PROVEAN, SIFT, and FATHMM-MKL. The frequency of the identified variants in the general population was checked using online databases such as EVS, 1000 Genomes, ExAC, gnomAD, and 2000+ in-house exomes. Finally, the amino acid conservation across different species was performed online using HomoloGene (NCBI; https://www.ncbi.nlm.nih.gov/homologene).

### Three-Dimensional Protein Modeling

The partial amino acid (663aa) sequence of ALKBH8-encoding protein was retrieved from the UniProt Knowledgebase database with accession number Q96BT7-1 in the FASTA format. In the absence of an experimentally known structure, comparative modeling is one of the most precise computational approaches to predict a consistent three-dimensional (3D) design from sequence information (Källberg et al., 2012). Due to the absence of an experimentally known structure for ALKBH8, its protein sequence was submitted to the I-TASSER server (Yang et al., 2015) for structure prediction. From models generated by I-TASSER, the model was selected on the basis of the I-TASSER evaluation score. The selected structure was optimized through 1,000 steps of steepest descent (Wardi, 1988) and 1,000 steps of conjugate-gradient (Dai et al., 2000) minimization by UCSF Chimera version 1.11 (Meng et al., 2006), and through Amber ff14SB force field, the 3D structure of mutated protein was generated by MODELLER (9.19) (Webb and Sali, 2014). The MODELLER assists in 3D structure prediction of the proteins by satisfying spatial restraints (Eswar et al., 2008). The model was selected based on the MODELLER evaluation score from the models generated by MODELLER.

### Model Evaluation

The recognition of errors in the experimental and theoretical models of protein structures is a significant problem in structural bioinformatics. Unfortunately, there is no single method that consistently and accurately predicts the errors in 3D structure (Melo et al., 1997). Therefore, different evaluation tools were used for the assessment of the protein structure. The model was further processed by RAMPAGE (Lovell et al., 2003) and ERRAT (Colovos and Yeates, 1993). RAMPAGE generates a Ramachandran plot for the assessment of models along with the distribution of residues in favored, allowed, and outlier regions. ERRAT generates a plot indicating the confidence and overall quality of the model.

## Results

### Clinical Assessment

In the present study, a consanguineous Pakistani family having a single affected individual was genetically and clinically evaluated. The affected individual (IV-1) was born at full term through vaginal delivery. At the age of 5–6 months, the parents observed developmental delay compared to other infants of the same age. At the age of 8–9 months, the index suffered from atonic seizures, diagnosed using EEG. The proband (IV-1) exhibited complex syndromic intellectual disability (ID) phenotypes, such as global developmental delay (GDD), hypotonia, speech impairment, seizure disorder, dysmorphic facial features (bulged eyes, prominent philtrum, high arched palate, large ears, thin upper lips, and lower nasal bridge), and bilateral postaxial polydactyly type B in the hands (Figure 1B). The parents were consanguineous, and the family pedigree depicts autosomal recessive inheritance. The parents of the affected individuals were healthy and did not reveal any phenotypic abnormality (Figure 1B; Table 1).

**TABLE 1:** Comparative clinical description of patients reported to date.

Clinical phenotypes	Family 1 (IV:13)	Family 2 (IV:12)	BAB13277	II:1	II:2	Present study (IV-1)
References	Monies et al. (2019)	Monies et al. (2019)	Saad et al. (2021)	Maddirevula et al. (2022)	Maddirevula et al. (2022)	
Sex	Male	Male	Male	Female	Female	Female
Origin	Saudi	Saudi	Egypt	Yemeni	Yemeni	Pakistani
Consanguinity	+	+	+	+	+	+
Pregnancy event	Normal	Normal	Normal	Normal	Normal	Normal
Global developmental delay	+	+	+	+	+	+
Speech delay	+	+	+	+	+	+
Mild–intellectual disability	+	+	+	+	+	+
Seizure	+	+	−	+	+	+
Hypotonia	−	+	+	−	−	+
Epilepsy	+	+	−	−	+	
Weak reflexes	+	−	−	−	−	+
Hyperactivity	Mild	Mild	Mild	−	−	Mild
Anxiety	−	−	−	−	−	−
Poor sleep	−	−	−	−	−	−
Repetitive tics	−	−	−	−	−	−
Major deficiency in memory and mathematical abilities	−	−	−	−	−	−
Age at last exam	12 years	16 years	3 years	7 years	10 years	14 years
Head circumference	Normal	Macrocephaly: 60 cm	49 cm	45 cm	−	51.5 cm
Height	Normal	Normal	83 cm	−	−	146 cm
Weight	Normal	Normal	11.3 kg	−	−	47 kg
Dysmorphic features	+	+	+	+	+	+
MRI brain	Normal. Rounded lesion in the right transverse sinuses	Macrocephaly	Under-opercularization and widening of Sylvian fissures, thinning of the genu of corpus callosum and delayed myelination of internal capsule.	Semilobar holoprosencephaly	Semilobar holoprosencephaly	Brain MRI revealed normal morphology
Skeletal survey	−	−	−	−	−	Polydactyly
Hearing test	−	−	−	Normal	Normal	Normal
Eye exam	−	−	−	Normal	Normal	Normal
Echocardiogram	−	Ventricular septal defect	−	Normal	Normal	Normal
Muscular issues	−	−	−	−	−	Normal
Chromosomal analysis	Normal	Normal	Normal	−	−	Normal
Genetic results	c.1660C > T; p.(Arg554*)	c.1794delC; p.(Trp599Glyfs*19)	c.1684delC; p.(Arg562Alafs*56)	c.1874G > A; p.(Arg625His)	c.1874G > A; p.(Arg625His)	c.1511G > C; p.(Trp504Ser)

Brain MRI revealed normal morphology and signal intensity of the supra- and infratentorial structures. No restriction diffusion was noted; however, a short corpus callosum was observed, with no intracranial susceptibility artifact. The ventricular system appeared normal in size and configuration, and no hydrocephalus was observed. Preserved flow voids of the major intracranial vessels and orbital cavities were within normal limits. The clinical description of all the reported cases has been summarized in Table 1.

### Whole Exome Sequencing and Sanger Sequencing

As the pedigree depicted autosomal recessive inheritance pattern, the filtration of the variants was performed according to the autosomal recessive pattern and preference was given to the homozygous variants, yet compound heterozygous variants were not ignored. Initial screening included all the reported ID genes. We focused only on pathogenic disease–causing non-synonymous (NS) variants causing nonsense, missense, splice site variants (SS), and frameshift coding insertions or deletions (indel).

WES identified a biallelic missense variant (c.1511G>C; p.(Trp504Ser)) in the exon 12 of the *ALKBH8* gene (NM_138775.3) located on chromosome 11q22.3, which segregated with the disease phenotype as validated using Sanger sequencing (Figure 1A). The identified homozygous variant (c.1511G>C; p.(Trp504Ser)) was not reported in the gnomAD/ExAC database containing 15,708 whole-genome sequences and 125,748 human exome sequences. A list of other filtered variants obtained after WES is given in Supplementary Material S1. The variant Trp504 was highly conserved across the different species (Figure 1D). The pathogenicity index of the identified mutation was calculated using various online available tools (Table 2).

**TABLE 2:** Pathogenicity of the identified *ALKBH8* variant (c.1511G>C; p.(Trp504Ser)).

Tool used	Pathogenicity prediction
SIFT	Damaging
PROVEAN	Damaging
REVEL	Pathogenic
MutationTaster	Disease causing
MutPred	Pathogenic
LRT	Deleterious
FATHMM-MKL	Damaging
EIGEN	Pathogenic
DEOGEN2	Damaging
DANN	0.9907
BayesDel (noAF)	Damaging
VarSome	Uncertain significance

### Three-Dimensional Protein Modeling

In this study, *in silico* methodology such as homology modeling for wild-type and mutant was carried out. The 3D structure of ALKBH8 was modeled by the I-TASSER server (Nayab et al., 2021). The predicted structure of ALKBH8 has a good degree of accuracy. Different evaluation programs assessed the final refined model. Using homology modeling, 3D models of wild-type and mutated ALKBH8 protein (p.(Trp504Ser)) were predicted and evaluated by online structure analysis tools. The Ramachandran plot indicated that approximately 89 and 93% of the residues in the wild-type and mutant structure lay in allowed regions of the torsion angles, respectively. The 3D structure of both wild-type and mutant of ALKBH8 was subjected into the ERRAT protein structure verification server (Figures 1E–G).

The missense mutations at Trp504 might affect the secondary structure of the protein. Our analysis revealed that Trp504 interacts with Ser469, Ile470, Leu500, Ile501, Met506, Glu507, and Tyr510 (Figures 1H,I). Although both valine and leucine contain nonpolar neutral side chains, the substitution of a smaller valine to the bigger leucine disturbed interaction with the surrounding amino acid residues differently. These new interactions, in turn, might potentially disrupt both protein secondary structure and function. Using DUET, SDM, ENCoM, and mCSM, we predicted that Trp504Ser mutation would cause a −2.929, −3.470, −1.169, and −3.115 kcal/mole change in ΔΔG, respectively, indicating that the variant would significantly destabilize the protein structure and hence disrupt its function (Figures 1E–I).

## Discussion

ID is a major developmental disorder defined by substantial limitations in intellectual presentation and reduced social, conceptual, and practical skills. It is likely to affect almost 1% of the overall population (Maulik et al., 2011). Although characterized as syndromic and non-syndromic forms, the non-syndromic forms of ID seem more common. At the same time, more patients with similar gene defects are identified, the syndromic features become ostensible. Although environmental causes have been reported, severe forms of ID are often linked with genetic defects in single genes (Najmabadi et al., 2011; Gilissen et al., 2014). Autosomal recessive ID (monogenic) has been excessively studied with the expansion of next-generation sequencing techniques, expanding genetic heterogeneity and phenotypic variability. According to Kochinke et al. (2016), 650 genes for ID have been characterized including ∼62% (autosomal recessive), ∼16% (X-linked), ∼3% (autosomal dominant), and 19% (*de novo*). The mutated genes in ID patients are involved in coding for highly diverse groups of proteins, suggesting crucial and nonredundant functions of these genes in multiple biological processes.

In the present study, using WES, we identified a homozygous missense variant (c.1511G>C; p.(Trp504Ser)) in exon 12 of the *ALKBH8* gene (NM_138775.3) that segregated with the disease phenotype. The identified variant is located in the highly conserved tRNA methyltransferase domain (MT) of the ALKBH8 protein located at the C-terminus (Figure 1C). The affected individual showed ID, facial dysmorphism, speech delay, and learning disability, which were the common features observed in the patients reported by Monies et al. (2019). Homozygous loss-of-function variants in the *ALKBH8* gene were first associated with syndromic ID (Monies et al., 2019). Saad et al. (2021) reported a novel *ALKBH8* variant in an Egyptian family associated with a neurodevelopmental disorder. We report, to the best of our knowledge, on the fourth novel variant in *ALKBH8* associated with syndromic ID. Our index’s phenotypes mirror the features reported previously (Monies et al., 2019; Saad et al., 2021). Previously, all three patients revealed a loss-of-function variant; however, more recently, a single family having two affected individuals revealed a biallelic missense variant (c.1874G>A; (p.Arg625His)) in the *ALKBH8* gene associated with syndromic ID (Maddirevula et al., 2022). Thus supporting our claim that missense variants in *ALKBH8* also cause syndromic ID in humans.

Novel gene discoveries have introduced a new emerging ID group associated with tRNA modifications, namely, the *ADAT3* gene, which edits adenosine to inosine at the wobble position 34 of mature tRNA (Alazami et al., 2013). Other genes include *NSUN2* (Abbasi-Moheb et al., 2012), *WDR4* (Shaheen et al., 2015), *TRMT10A* (Igoillo-Esteve et al., 2013), *TRIT1* (Yarham et al., 2014; Kernohan et al., 2017), *TRMT1* (Najmabadi et al., 2011), *ELP2* (Najmabadi et al., 2011), *PUS3* (Nøstvik et al., 2021), and *FTSJ1* (Freude et al., 2004).

Transfer RNAs (tRNAs) are vital molecules that participate in the final protein synthesis. Defects in tRNA modifications have been associated with different types of disorders such as myopathies, respiratory defects, metabolic (diabetes type II), mitochondrial disorders such as encephalopathy, myopathy, stroke-like episodes (MELAS), lactic acidosis, familial dysautonomia, and ID (Bednářová et al., 2017). The human brain is susceptible, and defects in tRNA modifications and mutations in specific genes involved in posttranscriptional modifications can be attributed to causing severe neurological disorders (Bednářová et al., 2017). tRNA modifications have been reported to cause ID phenotypes in humans, such as *PUS3* (OMIM 616283), which isomerizes uracil to pseudouridine in human tRNA homozygous pathogenic mutations in *PUS3* causing autosomal recessive ID (OMIM 617051; Nøstvik et al., 2021). In addition, methylation of tRNA at specific residues is performed by the *FTSJ1* (OMIM 300499), and mutations in these genes lead to non-syndromic X-linked mental retardation and ID (Guy et al., 2015).

Pathogenic disease–causing mutations in the tRNA modification genes exhibited severe clinical phenotypes. Understanding the proper function of these tRNA-modifying genes/enzymes will help us understand the precise biological roles, which might develop therapeutic strategies for this new class of disorders either by controlling tRNA gene expression or altering the low or highly expressed tRNA using CRISPR technologies (Khan et al., 2020).

In conclusion, we reveal that disease-causing biallelic missense variants in the *ALKBH8* are associated with an autosomal recessive form of syndromic neurological disorder. Our finding further supports the evidence that variants in ALKHB8 cause syndromic neurological diseases and add to the growing class of tRNA modification-related disorders.

## Data Availability

The data sets presented in this study can be found in online repositories. The names of the repository/repositories and accession number(s) can be found in the article/Supplementary Material.
